# Angiosarcoma of the Bladder: Case Report and Review of the Literature

**DOI:** 10.1100/tsw.2008.79

**Published:** 2008-05-22

**Authors:** Steve K. Williams, Rita L. Romaguera, Bruce Kava

**Affiliations:** ^1^ Department of Urology, Miller School of Medicine, University of Miami, Miami, FL, USA; ^2^ Department of Pathology, Miller School of Medicine, University of Miami, Miami, FL, USA

**Keywords:** angiosarcoma, bladder cancer, sarcoma, radiation induced malignancy

## Abstract

Our objective was to present a new case of angiosarcoma of the bladder after therapeutic radiation of the prostate, and discuss the treatment and clinical course of this rare tumor; the role of multimodality treatment is also discussed. We report a case of angiosarcoma of the bladder. Presentation, clinical course, and treatment were outlined and discussed. A MEDLINE search of all reported cases of angiosarcoma in the English language literature was performed. Thirteen previous cases of bladder angiosarcoma have been reported and three previous cases have been reported after therapeutic radiation. Hematuria was the most common presentation. Overall survival is poor, with 5-year survival rates at 35%. Longer-term survival has been demonstrated in patients who have had a multimodal approach to treatment, which combines radical surgery with chemotherapy and radiotherapy. Angiosarcoma of the bladder is a rare disease with overall poor prognosis. Optimal treatment has not been defined, but multimodality approaches appear to have a survival benefit.

Angiosarcoma is a rare vascular neoplasm that usually occurs in adults and may arise within virtually any region of the body. Most of these tumors are found in the head and neck region, or in the extremities. Visceral involvement is much rarer.

Predisposing factors that are thought to contribute to the development of angiosarcoma include ionizing radiation[1] and chemical agents, such as vinyl chloride[2]. Angiosarcoma of the bladder was first reported in 1907 by Jungano[3]. To our knowledge, only 13 such cases have been reported in the world-wide literature. We report a case of angiosarcoma of the bladder 10 years after therapeutic irradiation for prostate cancer.

## CASE REPORT

A 71-year-old, white male presented with a single episode of gross painless hematuria. His past medical history was significant for having undergone external beam radiotherapy for prostate cancer 10 years earlier. Physical examination revealed a palpable left pelvic mass. A computed tomography (CT) scan was performed, which confirmed the presence of a left-sided bladder wall mass with unilateral hydronephrosis. Cystoscopically, the tumor was found to arise from the left hemitrigone, and bimanual examination under anesthesia indicated a mass that appeared to be fixed to the pelvic sidewall. A transurethral resection of the tumor was performed and a left indwelling ureteral stent was placed. Pathology was consistent with epithelioid angiosarcoma infiltrating muscularis propria.

Subsequently, the patient underwent a radical cystoprostatectomy, pelvic lymph node dissection, and creation of an ileal conduit. Intraoperatively, there was a large extravesical mass that extended to and invaded the pelvic sidewall. A wide resection was carried out, revealing no residual gross tumor behind. The pathological examination revealed a mass with mucosal ulceration. The lesion involved the bladder wall and invaded through the detrusor muscle. It also involved the perivesical soft tissue, prostate, and seminal vesicle (see Fig. 1).

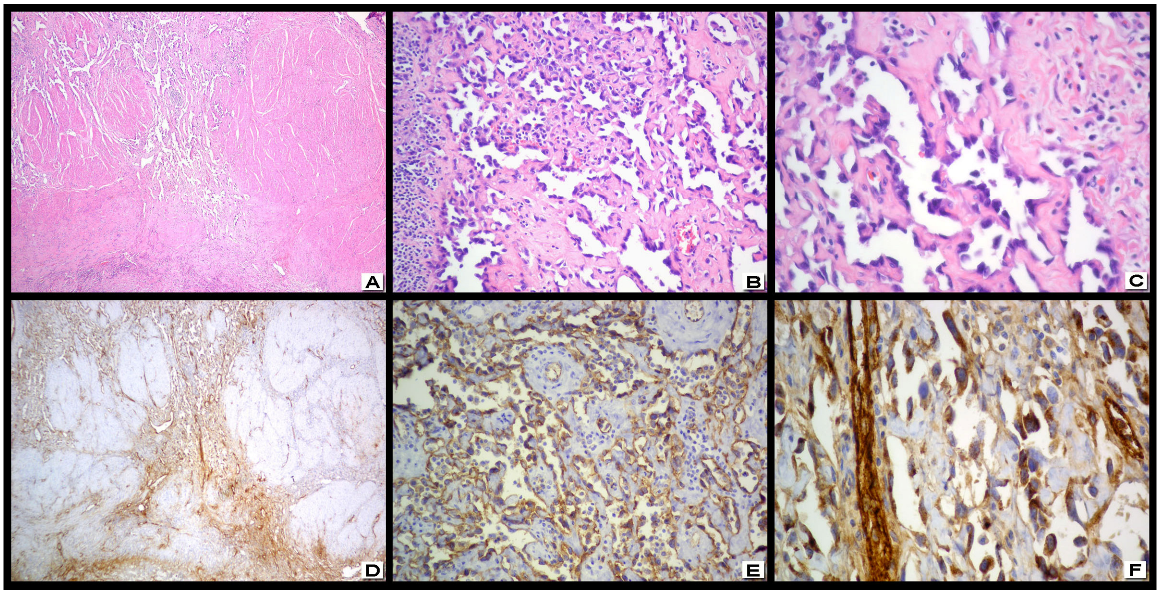

**FIGURE** 1. (A–C) Hematoxylin-eosin stained sections at 10, 20, and 40×, respectively, showing the bladder wall invaded by a vascular tumor, in which vascular channels are lined by atypical endothelial cells. (D–F) CD31 staining at 10, 20, and 40×, respectively, enhancing the vascular etiology of the tumor with the atypical endothelial cells staining for this endothelial marker.

The patient recovered from surgery, and was referred for adjuvant chemotherapy and radiation therapy. Despite a reasonably good appetite, he continued to lose weight postoperatively. He developed malignant ascites and bilateral lower extremity edema. A repeat CT scan 2 months following surgery revealed diffuse carcinomatosis. The patient subsequently opted for hospice care and died 3 months postoperatively.

## COMMENT

Nonurothelial tumors of the bladder account for less than 5% of all bladder tumors in the U.S.[4] Sarcoma represents the most common mesenchymal tumor of the bladder and generally shares an extremely aggressive biologic behavior. Angiosarcomas are a rare subtype of sarcoma and are highly malignant vascular tumors. Even though the pathologic features for bladder angiosarcomas are not well described, this patient's angiosarcoma showed the classic histology of freely anastomosing vascular channels lined by atypical endothelial cells (confirmed with immunohistochemical staining for CD31, CD34, and Factor VIII).

Very little is known regarding the natural history, optimal treatment, and prognosis of these tumors. Engel et al. reported on 10 cases with angiosarcoma of the bladder[5]. They found an almost universally poor prognosis, with an overall mean survival of only 8.5 months. These tumors are usually high grade and have reported 5-year survival rates of between 10 and 35%[6]. To date, 13 cases of bladder angiosarcomas have been reported. Three of these patients had an antecedent history of pelvic radiotherapy. Two cases were reported after radiation therapy for a gynecologic malignancy[7,8], while one appeared after therapeutic irradiation for prostate cancer[9]. None of these patients has an antecedent history of exposure to arsenic, thorium dioxide, or vinyl chloride.

The most common presentation of these patients is hematuria[10], while dysuria and flank pain are less common symptoms. There is a marked male predilection, with reports of male:female ratio of 8:1. The tumors tend to arise from all areas of the bladder. As with most sarcomas, the lung and the liver are common sites for metastases, with a hematogenous metastatic pattern the rule.

The development of a secondary malignancy is a recognized risk of radiation therapy[1,5,7,9]. In considering the diagnosis of a radiation-induced sarcoma, Cahan et al. suggested the following criteria: (1) the sarcoma should arise in the area previously subjected to irradiation, (2) a latent period (at least 7 years) must exist between the time of irradiation and development of the sarcoma, and (3) the sarcoma must be confirmed histologically[11]. The association of angiosarcoma with therapeutic radiation has been previously described[7,9].

The present case satisfies all of these criteria and, thus, can be considered radiation related. In addition to the direct oncogenic effect of ionizing radiation, prolonged cellular stimulation during repair of tissue damage resulting from radiation-induced ischemic change may play a role in development of angiosarcoma[12].

The management of these patients is surgical resection of the tumor with generous circumferential margins whenever possible. Radical cystectomy and ileal conduit was performed on this patient. Negative margins are crucial for cure and improved local control has been demonstrated in high-grade lesions with adjuvant radiotherapy[6]. Mark et al. reported an actuarial disease-free survival of 43% when adjuvant radiotherapy is given in angiosarcomas compared to 17% without. In general, randomized trials have failed to show a survival benefit for chemotherapy in the treatment of soft-tissue sarcomas[13]. Multimodal approaches with chemotherapy and radiotherapy have shown promising results in high-grade sarcomas of the head and neck[14]. Pazona et al. reported on long-term survival after multimodal therapy for bladder angiosarcoma[15]. They reported on a patient treated with radical cystectomy with adjuvant chemotherapy (mesna, doxorubicin, ifosfamide, and dacarbazine [MAID]) followed by pelvic irradiation. The patient died of a cardiac event 6 years after surgery with no evidence of disease at autopsy. Similarly, Engel et al. reported on a patient with primary angiosarcoma of the bladder treated with combined cystectomy, MAID chemotherapy, and external beam radiation[5]. They reported that at 32 months after diagnosis and initial treatment, the patient had no evidence of disease. Unfortunately, our patient had rapid disease progression after surgery, and was therefore not a candidate for adjuvant therapy.

## CONCLUSIONS

Angiosarcoma of the bladder is an extremely aggressive tumor with a short disease course. If an attempt at cure is feasible, a multimodal approach consisting of radical surgery followed by high-dose adjuvant radiotherapy and chemotherapy seems most effective.

## ACKNOWLEDGMENTS

Financial support was received from the Jackson Memorial Hospital Foundation.
